# Hepatitis B Virus Infection in Human Immunodeficiency Virus Infected Southern African Adults: Occult or Overt – That Is the Question

**DOI:** 10.1371/journal.pone.0045750

**Published:** 2012-10-01

**Authors:** Trevor G. Bell, Euphodia Makondo, Neil A. Martinson, Anna Kramvis

**Affiliations:** 1 Hepatitis Virus Diversity Research Programme, Department of Internal Medicine, University of the Witwatersrand, Johannesburg, South Africa; 2 Perinatal HIV Research Unit, University of the Witwatersrand, Johannesburg, South Africa; 3 Johns Hopkins University School of Medicine, Baltmore, Maryland, United States of America; University of Pretoria/NHLS TAD, South Africa

## Abstract

Hepatitis B virus (HBV) and human immunodeficiency virus (HIV) share transmission routes and are endemic in sub-Saharan Africa. The objective of the present study was to use the *Taormina* definition of occult HBV infection, together with stringent amplification conditions, to determine the prevalence and characteristics of HBV infection in antiretroviral treatment (ART)-naïve HIV^+ve^ adults in a rural cohort in South Africa. The presence of HBV serological markers was determined by enzyme linked immunoassay (ELISA) tests. HBV DNA-positivity was determined by polymerase chain reaction (PCR) of at least two of three different regions of the HBV genome. HBV viral loads were determined by real-time PCR. Liver fibrosis was determined using the aspartate aminotransferase-to-platelet ratio index. Of the 298 participants, 231 (77.5%) showed at least one HBV marker, with 53.7% HBV DNA^−ve^ (resolved) and 23.8% HBV DNA^+ve^ (current) [8.7% HBsAg^+ve^: 15.1% HBsAg^−ve^]. Only the total number of sexual partners distinguished HBV DNA^+ve^ and HBV DNA^−ve^ participants, implicating sexual transmission of HBV and/or HIV. It is plausible that sexual transmission of HBV and/or HIV may result in a new HBV infection, superinfection and re-activation as a consequence of immunesuppression. Three HBsAg^−ve^ HBV DNA^+ve^ participants had HBV viral loads <200 IU/ml and were therefore true occult HBV infections. The majority of HBsAg^−ve^ HBV DNA^+ve^ participants did not differ from HBsAg^+ve^ HBV DNA^+ve^ (overt) participants in terms of HBV viral loads, ALT levels or frequency of liver fibrosis. Close to a quarter of HIV^+ve^ participants were HBV DNA^+ve^, of which the majority were HBsAg^−ve^ and were only detected using nucleic acid testing. Detection of HBsAg^−ve^ HBV DNA^+ve^ subjects is advisable considering they were clinically indistinguishable from HBsAg^+ve^ HBV DNA^+ve^ individuals and should not be overlooked, especially if lamivudine is included in the ART.

## Introduction

Hepatitis B virus (HBV) and human immunodeficiency virus (HIV) share transmission routes and represent the two most important blood-borne pathogens in terms of prevalence, morbidity and mortality in sub-Saharan Africa, where both viruses are endemic. Of the 33.3 million adults and children living with HIV globally, 22.5 million reside in sub-Saharan Africa [1]. Moreover, it is estimated that 65% to 98% of populations in sub-Saharan Africa have been exposed to HBV and 8% to 20% are chronic carriers of HBV [2], far exceeding the 4% to 6% lifetime exposure rates and 0.2% to 0.5% carrier rates in regions of low endemicity. Thus, widespread co-infections are likely to occur, with 16% to 98% of HIV^+ve^ individuals in sub-Saharan Africa being carriers of HBV or showing exposure to HBV [3].

The progression of chronic HBV to cirrhosis, end-stage liver disease (ESLD), and hepatocellular carcinoma (HCC) is more rapid in HIV^+ve^ individuals than those with HBV alone [4], with a significant increase in hepatic-related mortality rates [5]. Furthermore, HBV co-infection negatively impacts on HIV outcomes [6]. Before the introduction of antiretroviral therapy (ART), the majority of HBV/HIV co-infected individuals were more likely to die from the clinical consequences of HIV than those of HBV [3]. However, since the introduction of ART, the disease profile has changed, with increases in the proportion of mortality attributed to HBV-associated ESLD [7]. Thus, HBV/HIV co-infection can potentially impact on the safety and effectiveness of ART, requiring an integrated approach for the appropriate management of co-infected individuals [8].

There is a paucity of comprehensive and standardized data describing HBV/HIV co-infection from southern African countries, where HIV prevalence is extremely high. Existing data show large discrepancies, with exposure rate to HBV in HIV^+ve^ South Africans varying from 28% to 99.8% and HBsAg prevalence ranging from 0.4% to 23% [9]–[18]. Differences can be attributed to different locations, study designs, laboratory measures and/or the composition of the study populations.

HIV infection has been implicated as a risk factor for the development of occult HBV infection (OBI) [12], defined by the *Taormina* expert panel as the “*Presence of HBV DNA in liver (with detectable or undetectable HBV DNA in the serum) of individuals testing HBsAg negative by currently available assays. When detectable, the amount of HBV DNA in the serum is usually very low (<200 IU/ml)*” [19]. Because liver biopsies are not commonly available, especially in resource-limited environments, OBI is usually detected by the analysis of sera [19]. Furthermore, the experts differentiate between true occult (HBV viral load <200 IU ml^−1^) and false occult where HBV DNA levels are comparable to those detected in HBsAg^+ve^ infection (overt) and are usually as a result of infection by HBV variants with S gene escape mutants, producing HBsAg that is not recognized by detection assays [19]. The clinical implications of OBI are unclear.

The prevalence of OBI in HIV infected individuals varies depending on the definition used, the sensitivity of the assay and the HBV viral loads [11]–[13], [16], [17]. Furthermore, studies performed outside Africa, in areas of low HBV and HIV endemicity, cannot necessarily be extrapolated to Africa because of differences in host factors, epidemiology, transmission patterns and genotypes of the viruses between the two regions.

The objective of the present study was to use the *Taormina* definition of OBI [19], together with stringent amplification conditions, to determine the prevalence and characteristics of HBV infection in ART-naïve HIV^+ve^ adults entering a rural cohort in Mpumalanga Province, which has a HIV prevalence of 15.4% [20]. No in-depth studies have been undertaken to determine the prevalence and characteristics of HBV/HIV co-infection in this province.

## Materials and Methods

### Subjects

A new rural cohort was established at Shongwe Hospital in Mpumalanga Province in South Africa and 298 ART-naïve, HIV^+ve^ adults were enrolled from July to November 2009. All had qualified for ART according to the then-current South African ART guidelines (CD4 counts <200 cells mm^−3^) [21] and were recruited while undergoing treatment-readiness counselling. Universal HBV vaccination at 6, 10, and 14 weeks of age was introduced into the South African Expanded Programme on Immunization (EPI) in 1995 and therefore none of the participants were likely to have received this vaccination and self-reported as unvaccinated. Clinical and demographic data (including ALT levels, CD4 T-cell count, age, sex, height and weight) were obtained from hospital records, the National Health Laboratory Services (NHLS) databases and the TherapyEdge-HIV (TE) ™ electronic patient record. All participants signed informed consent. The study was approved by the Human Research Ethics Committee (Medical) of the University of the Witwatersrand and Mpumalanga Department of Health Research Ethics Committee.

### Serology

The presence of HBsAg, anti-HBsAg and anti-HBcAg was determined for 298 sera using the Monolisa™ HBsAg ULTRA, HBsAb ULTRA and HBcAb PLUS ELISA kits (Bio-Rad, Hercules, CA), respectively. HBeAg and anti-HBe tests were performed on HBV DNA^+ve^ sera using the Monolisa™ HBeAg-Ab PLUS kit. Anti-HBcAg IgM was determined for 17 anti-HBc^+ve^ HBV DNA^+ve^ samples for which serum was available using the ARCHITECT® kit (Abbott Diagnostics, Wiesbaden, Germany). The M30-Apoptosense® ELISA (Peviva AB, Stockholm, Sweden) was used on all sera to quantify the apoptosis-associated cytokeratin 18Asp396 neo-epitope as a measure of hepatocyte apoptosis [22].

### Measurement of liver fibrosis

The aspartate aminotransferase (AST)-to-platelet ratio index (APRI) = (AST[/ULN]*100)/platelet count [10^9^ L^−1^], a noninvasive measure of liver fibrosis in patients with chronic HBV [23], was calculated for 163 subjects for whom AST levels and platelet counts were available. APRI indicates liver fibrosis only when liver disease has reached a severely advanced stage, with significant fibrosis defined as APRI≥1.5, and no fibrosis as APRI≤0.5 [24].

### Polymerase chain reaction (PCR)

DNA was extracted from 200 µl blood plasma with the QIAamp DNA Blood Mini Kit (QIAGEN Gmbh, Hilden, Germany) and eluted into 75 µl of best-quality water (BQW). Known positive and negative sera and BQW were used as controls for the extraction. Three regions of the HBV genome were amplified in a MyCycler™ thermocycler (Bio-Rad, Hercules, Ca, USA) using Promega Taq DNA polymerase (Promega, Madison, WI) (Table 1). To avoid cross-contamination and false positives, the precautions and procedures of Kwok and Higuchi [25] were strictly adhered to. DNA extraction, PCR, and electrophoresis were performed in physically separated venues.

**Table 1:** PCR primers and cycling parameters used for amplification of the three regions of the HBV genome.

Genome Region	Primer	Position^a^	Sequence	Cycles	Denaturation	Annealing	Extension	Size	Reference
Complete S PCR1F	2410(+)	2410-2439	5′-TCAATCGCCGCGTCGCAGAAGATCTCAATC-3′	40	94°C for 1 min	65°C for 1 min	72°C for 3 min	2126	[48]
Complete S PCR1R	1314(−)	1314-1291	5-′TCCAGACCXGCTGCGAGCAAAACA-3′						
Complete S PCR2F	2451(+)	2451-2482	5′-AATGTTAGTATTCCTTGGACTCATAAGGTGGG-3′	40	94°C for 1 min	66°C for 1 min	72°C for 3 min	2051	[48]
Complete S PCR2R	1280(−)	1280-1254	5′-AGTTCCGCAGTATGGATCGGCAGAGGA-3′						
Partial S PCR1F	231(+)	231-249	5′-TCACAATACCGCAGAGTCT-3′	40	94°C for 1 min	55°C for 1 min	72°C for 2 min	571	
Partial S PCR1R	801(−)	801-782	5′-AACAGCGGTATAAAGGGACT-3′						
Partial S PCR2F	256(+)	256-278	5′-GTGGTGGACTTCTCTCAATTTTC-3′	40	94°C for 1 min	55°C for 1 min	72°C for 2 min	541	[49]
Partial S PCR2R	796(−)	796-776	5′-CGGTATAAAGGGACTCACGAT-3′						
BCP PCR1F	1606(+)	1606-1625	5′-GCATGGAGACCACCGTGAAC-3′	40	94°C for 1 min	55°C for 1 min	72°C for 2 min	369	[48]; [50] b
BCP PCR1R	1974(−)	1974-1955	5′-GGAAAGAAGTCAGAAGGCCAA-3′						
BCP PCR2F	1653(+)	1653-1672	5′-CATAAGAGGACTCTTGGACT-3′	40	94°C for 1 min	55°C for 1 min	72°C for 2 min	307	[48]; [50] b
BCP PCR2R	1959(−)	1959-1941	5′-GGCAAAAAACAGAGTAACTCA-3′						

a Nucleotide position of HBV *adw* genome (GenBank accession number (AY233276), where position 1 is the *EcoR*I cleavage site.

b Modifications underlined.

(+) and “F” indicate forward (sense) direction; (−) and “R” indicate reverse (anti-sense) direction; PCR1 indicates first round PCR; PCR2 indicates second round PCR.

### Real-time PCR quantification of HBV DNA

PCR primers, HBV-Taq1 and HBV-Taq2 covering a region of the S gene (321 to 401 from the *EcoR*I site) with a FAM/TAMRA labelled TaqMan BS-1 probe [26] were used to quantify HBV DNA in an ABI 7500 Real Time PCR System (Applied Biosystems, Foster City, Ca, USA). A serial dilution of cloned plasmid DNA containing a single genome of HBV DNA, with concentrations ranging from 2×10^1^ to 2×10^11^ IU ml^−1^, was used as template to generate the standard curve. The second WHO International Standard for HBV Nucleic Acid Amplification Techniques (product code 97/750 National Institute for Biological Standards and Controls (NIBSC); Hertfordshire, UK), which has a final concentration of 10^6^ IU ml^−1^ was used as the internal standard. The standard curve, blank, positive and negative controls, and samples were all tested in duplicate. The measured IU/ml for each reaction was calculated using the Ct (cycle threshold) value of each PCR interpolated against the linear regression of the standard curve. The lower detection limit of our assay is ∼20 IU ml^−1^. The conversion formula of IU = copies/4.7 was used [11], [27].

### Statistical analysis

Clinical data were inspected visually. As all continuous variables showed a skewed distribution, the Mann-Whitney U test (Wilcoxon rank-sum test) was used to compare samples. Chi-squared and Fisher's exact test were used to compare categorical variables. Exhaustive multivariate logistic regression analyses were performed. The R statistical language was used throughout [28].

## Results

### Serological and nucleic acid testing for HBV

The study group consisted of 298 adults (114 men and 184 women) with median age, CD4 count and BMI of 34 years, 147 cells mm^−3^ and 22 kg m^−2^, respectively. Men were older than women and had lower CD4 counts (Table 2).

**Table 2:** Characteristics of treatment-naïve HIV-infected adults: comparison of HBV DNA^+ve^ *versus* HBV DNA^−ve^ individuals^a^.

Characteristic	All participants (n = 292)			HBV DNA^+ve^ participants (n = 71)			HBV DNA^−ve^ participants (n = 221)			Significance – p-values^b^			
	All	Male[A]	Female[A]	All[D]	Male[B]	Female[B]	All[D]	Male[C]	Female[C]	A	B	C	D
**Numbers (%)**	292^c^	113(39%)	179(61%)	71	30(42%)	41(58%)	221	83(38%)	138(62%)	n/a	0.54	0.72	n/a
**Age in years**	34(28–41)	36(32–43)	32(27–40)	35(28–41)	37(34–47)	31(25–39)	33(28–41)	35(32–41)	32(27–40)	<0.0001	0.01	0.002	0.42
**Age at sexual debut in years** d	17(16–19)	17(16–20)	17(16–18)	17(16–18)	17(16–19)	16(15–18)	17(16–19)	17(16–20)	17(16–18)	0.035	0.12	0.11	0.36
**Lifetime sexual partners** e	3(1–5)	4(2–8)	2(1–4)	3(2–5)	4(2–8)	3(2–5)	3(1–5)	4(2–8)	2(1–3)	<0.0001	0.34	<0.0001	0.025
**BMI in kg m^−2^**	22(20–25)	21(19–24)	23(20–26)	22(20–24)	21(20–23)	22(20–25)	22(20–26)	21(19–24)	23(20–26)	0.0051	0.37	0.007	0.43
**ALT in U L^−1^**	21(12–32)	27(17–38)	18(11–27)	23(15–37)	31(19–59)	20(12–28)	20(12–31)	26(17–37)	17(11–27)	<0.0001	0.027	<0.0001	0.12
**Apoptosense in U L^−1^**	110(89–150)	112(91–150)	109(89–150)	114(91–158)	117(91–160)	114(91–152)	109(89–142)	111(92–141)	109(88–147)	0.63	0.96	0.68	0.34
**CD4 cells mm^−3^**	147(76–196)	99(49–171)	179(104–222)	148(74–199)	104(60–171)	180(103–245)	144(76–194)	98(46–169)	177(104–215)	<0.0001	0.006	<0.0001	0.83

a all values expressed as “Median (Interquartile Range)”.

b comparing the columns marked by UPPER case letter; statistical significance is indicated by underlining.

c 6 of the 298 participants were not included in the analyses because they lacked a complete data set.

d 9 samples without data points omitted.

e 2 samples without data points omitted.

The 298 participants were classified into five serogroups: 28 (9.4%) HBsAg^+ve^, 57 (19.1%) isolated anti-HBc^+ve^, 123(41.3%) anti-HBc^+ve^anti-HBs^+ve^, 11 (3.7%) anti-HBs^+ve^ alone and 79 (26.5%) serologically^−ve^ for HBV. Six percent of men (7/114) were anti-HBs^+ve^ alone compared to 2% (4/184) of women (p<0.05). The HBV serologically^−ve^ participants were significantly younger than most HBV serologically^+ve^ groups and had significantly fewer lifetime sexual partners than those with isolated anti-HBs^+ve^ (p<0.05). There was no significant difference in serologically-negative and -positive individuals in terms of CD4 counts, age of sexual debut, BMI, ALT and Apoptosense® levels. Only five participants were HBeAg^+ve^ and they did not differ from HBeAg^−ve^ individuals in either demographic or clinical features.

**Figure 1:**
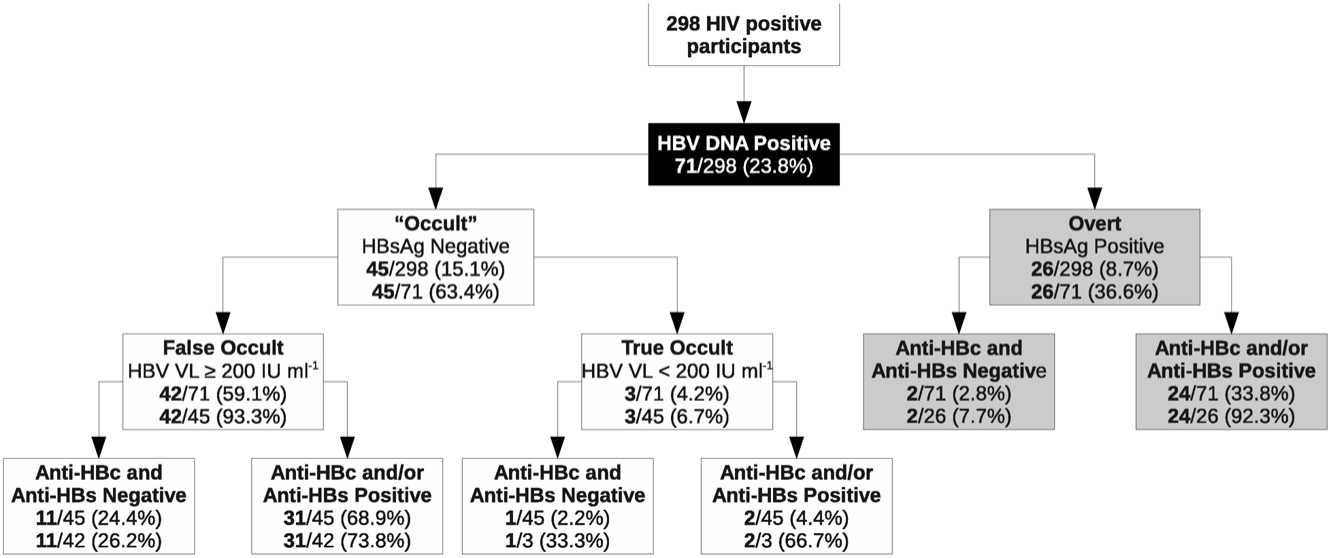
Serological and DNA markers for HBV detected in 71 of 298 HIV^+ve^ participants. Overt refers to HBsAg^+ve^ and “occult” to HBsAg^−ve^. According to the *Taormina* definition, false occult infections are HBsAg^−ve^ with HBV viral load (VL) ≥200 IU ml^−1^ and true occult infections are HBsAg^−ve^ with HBV VL <200 IU ml^−1^ [19].

Screening for HBV DNA was carried out using primers targeting three non-overlapping regions of the HBV genome (Table 1). A sample was considered to be HBV DNA^+ve^, only if at least two regions amplified. Sixty-seven of 298 participants (22.5%) lacked HBV DNA and all HBV serological markers, ruling out HBV exposure and/or infection and with no antibodies against HBV would be susceptible to acquiring HBV infection. The remaining 231/298 (77.5%) showed at least one marker for HBV, with 160/298 (53.7%) HBV DNA^−ve^ (resolved) and 71/298 (23.8%) HBV DNA^+ve^ (current) [26/298 (8.7%) HBsAg^+ve^ (overt): 45/298 (15.1%) HBsAg^−ve^ (“occult”)] (Figure 1).

**Figure 2:**
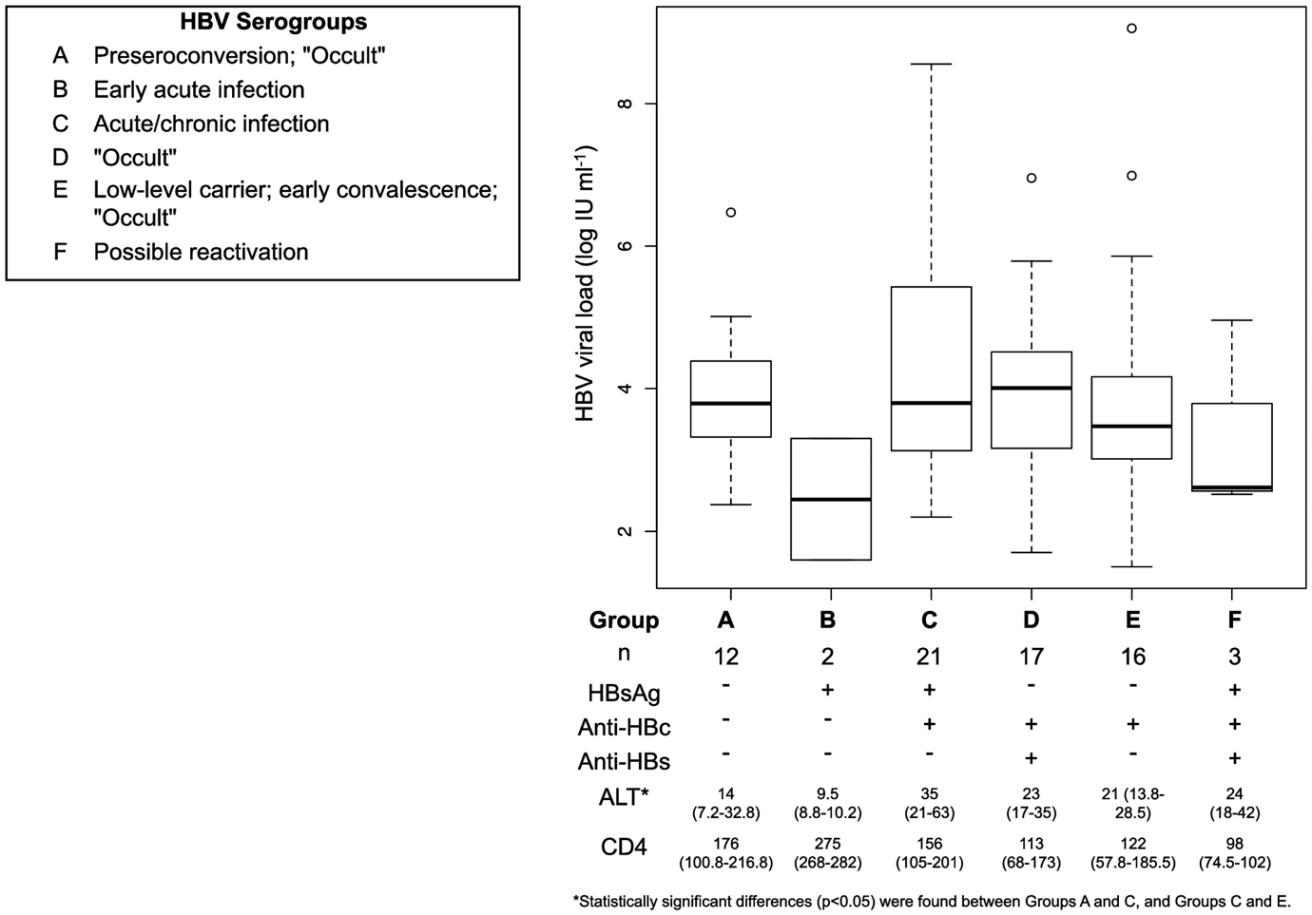
Box and whisker plot of HBV viral loads of the 71 HBV DNA^+ve^ participants separated into the six serological groups (A to F), interpreted according to Hollinger (2008) with modifications [35]. “n” indicates the number of participants in each group. ALT and CD4 cell counts for each group are indicated in the table below the plot as “Median (Interquartile Range)”. Viral loads and CD4 cell counts did not differ significantly between the six serological groups. The five HBeAg^+ve^ participants belonged to serological group C.

Of the entire group of 298, 26 (8.7%)/28 (9.4%) HBsAg^+ve^ participants were HBV DNA^+ve^ and together with the 45 (15.1%) HBsAg^−ve^ HBV DNA^+ve^ participants were classified into 6 serogroups (Figure 2). Within the HBsAg^−ve^ groups, the frequency of HBV DNA was significantly higher in anti-HBc^+ve^ alone individuals (16/57; 28.1%) compared to those anti-HBc^+ve^anti-HBs^+ve^ (17/123; 13.8%) (p<0.05). The relative risk of an HBsAg^−ve^ individual, who was anti-HBc^+ve^ alone, being HBV DNA^+ve^ was twice as high as that of one with anti-HBc^+ve^anti-HBs^+ve^. The frequency of HBV DNA in the serologically^−ve^ group was not significantly different to that in the anti-HBc^+ve^ alone or anti-HBc^+ve^anti-HBs^+ve^. Moreover, HBV DNA was not detected in any of the 11 isolated anti-HBs^+ve^ individuals. Sufficient serum was available to test for anti-HBc IgM in 17 of 57 anti-HBc^+ve^ HBV DNA^+ve^ participants and all tested negative. Only three HBsAg^−ve^ HBV DNA^+ve^ participants had viral loads <200 IU ml^−1^, thus meeting the *Taormina* criterion for true OBI [19]. These participants had serological patterns of groups A, D and E, respectively (Figure 2). All other HBsAg^−ve^ HBV DNA^+ve^ individuals had HBV viral loads >200 IU ml^−1^.

### Comparison of demographic and clinical characteristics between HBV DNA^+ve^ and HBV DNA^−ve^ groups

Visual inspection of plots and linear regression models of each of the continuous variables in Tables 2 and 3 (age, age at sexual debut, lifetime sexual partners, BMI (body mass index), ALT, Apoptosense, CD4 cell count, HBV viral load) against each other, for HBV DNA^+ve^ versus HBV DNA^−ve^, and HBsAg^+ve^ versus HBsAg^−ve^ groups, revealed no significant correlation.

**Table 3:** Characteristics of treatment-naïve HIV-infected adults: comparison of HBsAg^+ve^HBV DNA^+ve^ *versus* HBsAg-ve HBV DNA^+ve^ individuals^a^.

Characteristic	HBV DNA^+ve^ participants (n = 71)			HBsAg^+ve^HBV DNA^+ve^ (n = 26)			HBsAg-ve HBV DNA^+ve^ (n = 45)			Significance–p values^b^			
	All	Male[A]	Female[A]	All[D]	Male[B]	Female[B]	All[D]	Male[C]	Female[C]	A	B	C	D
**Numbers (%)**	71	30(42%)	41(58%)	26	13(50%)	13(50%)	45	17(38%)	28(62%)	n/a	0.42	0.54	n/a
**Age in years**	35(28–41)	37(34–47)	31(25–39)	34(27–28)	35(33–39)	29(25–35)	36(29–45)	38 (35–47)	34(28–40)	0.01	0.07	0.035	0.14
**Age at sexual debut in years** c	17(16–18)	17(16–19)	16(15–18)	17(16–20)	18(16–22)	17(15–18)	16(15–18)	17(16–18)	16(15–18)	0.12	0.11	0.66	0.23
**Lifetime sexual partners** d	3(2–5)	4(2–8)	3(2–5)	3(1–6)	2(1–6)	3(1–5)	4(2–5)	4(2–10)	3(2–5)	0.34	0.98	0.17	0.38
**BMI in kg m^−2^**	22(20–24)	21(20–23)	22(20–25)	23(20–25)	22(20–23)	25(20–27)	21(20–23)	21(20–22)	22(20–24)	0.37	0.29	0.64	0.30
**ALT in U L^−1^**	23(15–37)	31(18–59)	20(12–28)	30(19–59)	35(22–60)	21(12–39)	20(13–32)	27(15–37)	19(13–25)	0.027	0.29	0.12	0.07
**Apoptosense U L^−1^**	114(91–158)	117(91–160)	114(91–152)	113(84–156)	110(75–150)	114(91–161)	114(94–158)	121(96–188)	114(91–142)	0.96	0.27	0.37	0.62
**CD4 cells mm^−3^**	148(74–199)	104(60–171)	180(103–245)	152 (101–202)	125 (98–184)	179 (132–255)	147(68–196)	87(57–165)	181(91–229)	0.006	0.13	0.016	0.52
**LogHBVVL** e	3.72(3.04–4.41)	3.76(2.95–4.47)	3.61(3.08–4.35)	3.64(2.73–4.80)	4.14(2.74–5.44)	3.22(2.54–4.01)	3.78(3.15–4.35)	3.74(2.99–4.28)	3.81(3.19–4.44)	0.66	0.14	0.59	0.74

a all values expressed as “Median (Interquartile Range)”.

b comparing the columns marked by UPPER case letter; statistical significance is indicated by underlining.

c 9 samples without data points omitted.

d 2 samples without data points omitted.

e “LogHBVVL” indicates the log of the HBV viral load rounded to two decimal places [A Mann-Whitney U test was used on the untransformed viral loads (IU ml^−1^)].

A multiple logistic regression model was used to determine predictors of HBV DNA positivity. In this model, only ALT levels were significant when all variables were included (p<0.05; OR = 1.01; 95% CI: 1.002–1.020). When the data were split according to gender, number of lifetime sexual partners was the only predictor in the females (p<0.05; OR = 1.16; 95% CI: 1.01–1.36) and ALT in the males (p<0.05; OR = 1.02; 95% CI: 1.004–1.030).

As shown in Table 2, the only variable that differentiated the HBV DNA^+ve^ and HBV DNA^−ve^ groups was number of lifetime sexual partners (p<0.05). Regardless of whether they were HBV DNA^+ve^ or HBV DNA^−ve^, males were older than females, had a higher ALT and lower CD4 count. In the whole cohort and the HBV DNA^−ve^ group, females had a higher BMI (p<0.05) and fewer sexual partners than males (p<0.05). These differences were not seen in the HBV DNA^+ve^ group. The age of sexual debut was significantly different only when comparing males and females in the whole cohort.

### Comparison of demographic and clinical characteristics between HBsAg^+ve^ HBV DNA^+ve^ and HBsAg^−ve^ HBV DNA^+ve^ groups

Data from the HBV DNA^+ve^ participants were examined by logistic regression for predictors of HBV DNA-positivity in the absence of HBsAg. Only increasing age was weakly significant. The female subset showed that age was a significant predictor (p<0.05; OR = 1.28; 95% CI: 1.06–1.72). No predictors in the male subset were significant.

In the HBsAg^−ve^ HBV DNA^+ve^ group, men were older and had significantly lower CD4 cell counts compared to females (p<0.05). Although the difference in ALT levels between the HBsAg^+ve^HBV DNA^+ve^ and HBsAg^−ve^HBV DNA^+ve^ groups did not reach statistical significance (Table 3), individuals who were HBsAg^+ve^anti-HBc^+ve^ HBV DNA^+ve^ [group C] had significantly higher ALT levels compared to individuals who were either serologically^−ve^ HBV DNA^+ve^ [group A] (p<0.05) or anti-HBc^+ve^HBV DNA^+ve^ [group E] (p<0.05) (Figure 2). There was no significant difference between the HBsAg^+ve^ and HBsAg^−ve^ DNA^+ve^ groups when ALT levels were coded into binary groups: >29 U/L for males and >19 U/L for females. HBV viral loads did not differ significantly between HBsAg^+ve^ and HBsAg^−ve^ groups (Table 3).

### Measurement of liver fibrosis using APRI score

Ten percent of 163 individuals, for which data were available, had elevated APRI scores (≥1.5), representing advanced fibrosis: 7.94% (10/126) HBV DNA^−ve^ [5.3% (2/38) seronegative and 9.1% (8/88) seropositive] and 16.2% (6/37) HBV DNA^+ve^ [26.7% (4/15) HBsAg^+ve^HBV DNA^+ve^ and 9.1% (2/22) HBsAg^−ve^ HBV DNA^+ve^]. The frequency of liver fibrosis was significantly higher in HBsAg^+ve^HBV DNA^+ve^ individuals compared to seronegative HBV DNA^−ve^ ones (p<0.05), but not to seropositive HBV DNA^−ve^ ones (p = 0.07). There was no significant difference between the HBsAg^+ve^ and HBsAg^−ve^ HBV DNA^+ve^ groups.

## Discussion

In this group of 298 southern African ART-naïve HIV^+ve^ individuals, 231 participants had at least one HBV marker, giving an overall exposure to HBV of 77.5%, comparable to that in HBV monoinfected individuals [2]. In addition, almost one quarter of the group was HBV DNA^+ve^ (Figure 1) of whom almost two thirds were HBsAg^−ve^. Direct comparison with other South African ART-naïve HIV^+ve^ cohorts is difficult because of the different markers were used to measure exposure. In Limpopo Province, exposure to HBV, measured by anti-HBc and/or anti-HBs positivity, was 28.2% in a rural cohort [17] and 39.2% in anti-natal HIV^+ve^ women [16]. This differs from the 63% HBV exposure rate (measured by at least one marker: HBsAg, anti-HBs or anti-HBc) found in a rural-urban HIV^+ve^ cohort in Limpopo [13] and the much higher exposure rate of 99.8% in hospital-admitted HIV^+ve^ patients [12]. In Gauteng Province, a 47% exposure was seen in an urban HIV^+ve^ cohort where ∼15% were HBV-positive as follows: 4.8% HBsAg^+ve^ [10], 7.6% anti-HBc^+ve^HBV DNA^+ve^ [11] and 2.4% serologically^−ve^ HBV DNA^+ve^ [27].

The 9.4% HBsAg prevalence was comparable to that reported for some HIV^+ve^ South African cohorts: 6.2% in anti-natal women in Limpopo Province [16]; 7.1% in rural Eastern Cape (6.6% in ART-treated *versus* 8.8% in ART-naïve, p>0.05) [9]; and 6% in a country-wide study of treatment-naïve HIV^+ve^ military personnel and their family members [14]. On the other hand, the HBsAg prevalence was higher than the 0.4% in another rural cohort in Limpopo Province [17], double the 4.8% in a Gauteng urban cohort [10], but lower than the 11.3% in hospital-admitted Limpopo Province patients [12], the 19.7% in miners [15] and the 22.9% from a rural-urban cohort in Limpopo Province [13]. This difference in HBsAg prevalence correlates with the variations reported in HBV monoinfected individuals from different locales [16], [29], [30].

Regardless of whether they were HBV DNA^+ve^ or HBV DNA^−ve^, males were older, had higher ALT levels and lower CD4 counts than females (Table 1). These differences are because males tend to come for treatment later than females [31]. In the cohort as a whole and in the HBV DNA^−ve^ group, males had significantly more partners than females, with BMI significantly lower. In the HBV DNA^+ve^ group, these factors did not differ between the genders. The only factor differentiating the HBV DNA^+ve^ versus HBV DNA^−ve^ participants was the number of lifetime sexual partners (Table 2), suggesting sexual transmission of HBV and/or HIV. It is plausible this mode of transmission may result in a new HBV infection, superinfection and re-activation as a consequence of immunesuppression. Twenty percent of the 71 HBV DNA+ve participants had possible markers of recent infection: 12 serologically^−ve^ HBV DNA^+ve^ and 2 HBsAg^+ve^ HBV DNA^+ve^ (Figure 2). Of the 17 anti-HBc^+ve^ HBV DNA^+ve^ sera tested for anti-HBc IgM, none were positive.

The HBV serology of the cohort, the high frequency of HBeAg-negativity, the absence of anti-HBc IgM and the relatively low HBV viral loads (Figure 2) reflect the natural history of HBV infection in sub-Saharan Africa, where most individuals are infected at childhood by horizontal transmission [2]. This means that most individuals have been exposed to HBV, and are protected by anti-HBV antibodies, by the time they become sexually active and acquire HIV. All isolated anti-HBs^+ve^ participants were HBV DNA^−ve^ and the presence of anti-HBs with anti-HBc reduced the risk of being HBV DNA^+ve^. None of the participants had received HBV vaccination.

The HBsAg prevalence in this HIV^+ve^ cohort was not different to HIV^−ve^ cohorts [2], [3]. This differs from observations in areas of low HBV and HIV endemicity, where HBV and HIV are acquired simultaneously and therefore HBsAg prevalence in HIV^+ve^ individuals is significantly higher than in HIV^−ve^ individuals [3]. Only four participants in the present study were HBsAg^+ve^ alone: two were HBV DNA^+ve^, whereas the other two were HBV DNA^−ve^, even after repeated attempts to amplify HBV DNA, possibly indicating low viral loads undetectable by PCR. This might reflect the process of natural HBsAg clearance [32]. Although immune suppression by HIV may lead to the HBsAg^+ve^anti-HBc^−ve^ profile [33], this is unlikely in these two cases, considering that ∼59% of the participants were anti-HBc^+ve^, with a third of these having isolated anti-HBc. Moreover, HIV^+ve^ patients with CD4<100 cells mm^3^ are more likely to have isolated anti-HBc [34].

HBV DNA without HBsAg was detected in 15.1% of the participants (Figure 1). This is within the 8% to 18% range for South African HIV^+ve^ cohorts but again direct comparison is complicated by differences in study design [11]–[13], [16], [17]. Twelve participants were serologically^−ve^ HBV DNA^+ve^, which can occur before the appearance of HBsAg, in the preseroconversion phase (indicating a recent infection), or at the tail end of the infection [35]. Anti-HBsAg seroconversion, in the presence or absence of anti-HBc, decreased the relative risk of being HBV DNA^+ve^ in the HBsAg^−ve^ group. This agrees with findings in HBV monoinfected [36] and in HBV/HIV coinfected individuals [37].

There was no difference in the demographics of the HBV DNA^+ve^ subjects, with and without HBsAg (Table 2). In the presence of HBsAg, there was no difference between males and females, whereas in the absence of HBsAg, males were older and had lower CD4 counts than females. Thus older males with lower CD4 counts are more likely to be HBsAg^−ve^ HBV DNA^+ve^. Lower CD4 counts have been associated with HBsAg^−ve^ viremia regardless of gender [37], however the median CD4 counts in that study were relatively higher (316 cells mm^−3^ versus 147cells mm^−3^ in the present study) [37].

In agreement with other studies [38], [39], there were similar ALT levels in HBsAg^+ve^ and HBsAg^−ve^ HBV DNA^+ve^ participants and between HBV DNA^+ve^ and HBV DNA^−ve^ participants. The absence of transaminitis is as a result of the immunosuppressed state of the HIV^+ve^ subjects. Immunosuppression causes HBV reactivation and can lead to high viremia without clinical manifestation [40]. The APRI score was used to compare the frequency of liver fibrosis in the HBV^+ve^ *versus* HBV^−ve^ participants. The frequency of liver fibrosis was significantly higher in HBsAg^+ve^ HBV DNA^+ve^ individuals compared to seronegative HBV DNA^−ve^ ones, but not relative to seropositive HBV DNA^−ve^ ones. It is intriguing that there was no difference in the frequency of liver fibrosis between HBV DNA^+ve^ individuals, with and without HBsAg.

The reactivation of an infection, which originated in childhood, can explain why no significant difference was seen in the HBV viral loads between the HBsAg^+ve^ and HBsAg^−ve^ participants (Table 2), nor between the different serological groups (Figure 2). Following HIV infection, HBV can reactivate in anti-HBs^+ve^ only individuals, with and without the reappearance of HBsAg [41]. Group F, which had the lowest CD4 count of <100 cells mm^−3^, and by inference was the most immunosuppressed, was HBsAg^+ve^ anti-HBc^+ve^ anti-HBs^+ve^ HBV DNA^+ve^ with a viral load >10^2^ IU ml^−1^ (Figure 2). Spontaneous reverse seroconversion, where anti-HBs disappears and HBsAg reappears can also occur in the presence of CD4 counts <200 cells mm^−3^ [42]. Although HBV viral loads have been shown to be higher in HBV^+ve^ HIV^+ve^ individuals compared to HBV^+ve^ ones [43], the HBV viral loads detected in the present study were comparable to those detected in HBV mono-infected individuals [44]. This is probably because the majority of individuals were infected with subgenotype A1 [45], which is characterized by relatively low viral loads in mono-infected individuals compared to other genotypes or subgenotypes [44].

Only three HBsAg^−ve^ HBV DNA^+ve^ patients had HBV loads <200 IU ml^−1^, meeting the *Taormina* criterion for OBI. Thus the majority of HBsAg^−ve^ HBV DNA^+ve^ would be classified as false “occult” [19]. It is possible that immunosuppression precludes true occult HBV infection. Because the majority of HBsAg^−ve^ HBV DNA^+ve^ (“occult”) participants did not differ from HBsAg^+ve^ HBV DNA^+ve^ (overt) participants in terms of viral loads, CD4 counts, ALT levels and frequency of liver fibrosis, it may be more accurate to refer to these HBV infections as *HBsAg-covert* (HBsAg-***c***ryptic ***o***vert) instead of false “occult” [19].

HIV infection was demonstrated to be a risk factor for HBsAg^−ve^ HBV infection [12], and pre-S mutations preventing HBsAg secretion [46], ‘a’ determinant mutations leading to detection escape and overlapping polymerase mutations affecting replication, may be responsible for this. This possibility was investigated and is presented in a follow-up paper, where 12 of 13 HBV S region sequences, from HBsAg^−ve^ participants, had pre-S and/or S mutations [45]. Another possible explanation for HBsAg-negativity may be that HIV co-infection prevents HBsAg secretion, as shown in co-infected hepatic cell lines [47].

Despite the possible limitations of this study, including its cross-sectional nature, the absence of HIV viral loads, no HBV mono-infected patients and patients with higher CD4 counts for comparison, a number of important conclusions can be reached. The number of lifetime sexual partners was the only factor differentiating HBV DNA^+ve^ and HBV DNA^−ve^ infections, suggesting sexual transmission of HBV and/or HIV. HBV^+ve^ HIV^+ve^ individuals were found to have significantly higher lifetime sexual partners than HBV-monoinfected individuals [18]. HBV infection in HIV^+ve^ individuals was predominantly HBsAg^−ve^, which did not differ significantly from HBsAg^+ve^ infections in terms of viral loads, CD4 counts, ALT levels and frequency of liver fibrosis.

The detection of HBV DNA in the absence of HBsAg in this and other South African studies [11]–[13], [17] has important implications for the clinical management of HIV in sub-Saharan Africa, where the burden of HBV/HIV co-infection is disproportionately high (24% in this study). Although the World Health Organization recommends that ART be initiated in HBV/HIV co-infected individuals irrespective of CD4 count, in South Africa we face a number of challenges. The most recent South African guidelines recommend initiation of treatment of patients with CD4 counts <350 cells mm^−3^ and HBsAg testing if ALT levels exceed 100 U L^−1^. Considering that the highest median ALT levels (IQR) of 30 (19–59) U L^−1^ were found in the HBsAg^+ve^ HBV DNA^+ve^ group (Table 3), which also had the highest frequency of advanced fibrosis, this cut-off value is inappropriate. Moreover, 65% of the 71 participants, who were HBV^+ve^ HIV^+ve^, lacked HBsAg and HBV could only be detected by nucleic acid testing, which is unaffordable in resource-limited environments. Although the clinical significance of HBsAg^−ve^ infection is under debate [32], it is imperative that HBV/HIV co-infection is detected before ART initiation, especially because lamivudine remains in two of the three drug regimens currently provided by the South African government and HBV can develop resistance to lamivudine. To determine the clinical relevance of *HBsAg-covert* HBV infection in our setting, prospective studies following ART initiation are in progress.
